# Cr-Triggered FCC Ti-Rich Precipitation and Its Effects on the Mechanical and Oxidation Performance of TiVNbTa Refractory High-Entropy Alloys

**DOI:** 10.3390/ma19183886

**Published:** 2026-09-11

**Authors:** Shaomin Luo, Juan Li

**Affiliations:** 1 School of Aerospace Engineering, Guizhou Institute of Technology, Guiyang 550025, China; 2 School of Materials and Energy Engineering, Guizhou Institute of Technology, Guiyang 550025, China

**Keywords:** refractory high entropy alloy, Cr content, FCC Ti-rich precipitate, mechanical property, oxidation behavior

## Abstract

This study investigates the effects of Cr content on the microstructure, room-temperature and 600 °C mechanical properties and high-temperature oxidation behavior of TiVNbTaCr_x_ (x = 0, 0.25, and 0.5). Cr addition significantly intensified Ti segregation in the interdendritic regions during solidification, inducing the formation of an Face-Centered Cubic (FCC) Ti-rich precipitate phase. As the Cr content increases from 0 to 0.5, the Ti-rich phases evolve from a straw-like morphology to a blocky morphology, and the mechanical properties at room temperature continue to decrease. Fracture analysis reveals that cracks initiate at grain boundaries associated with the FCC Ti-rich phase, leading to a transition from ductile to brittle fracture in the Cr-containing alloys. At 600 °C, all Cr-containing alloys exhibit elongations below 0.20%, indicating that the precipitation of the FCC Ti-rich phase severely degrades the deformation capability of the alloys at 600 °C. Oxidation tests demonstrate that Cr addition provides only a marginal beneficial effect on the mass gain rate during the initial stage (0–10 h at 600 °C), while the Cr-containing alloys continue to gain mass upon prolonged exposure due to the reduced integrity of the oxide film. The oxide scale consists primarily of (Ta, Nb)_9_VO_25_, and no protective Cr_2_O_3_ scale was observed under the present experimental conditions. In summary, within the composition range studied, the FCC Ti-rich phase precipitates at Cr contents of 5.88 and 11.11 at.%, resulting in the fragmentation of the body-centered cubic lattice (BCC) matrix continuity and a comprehensive deterioration of mechanical properties at both room temperature and 600 °C, while failing to improve the oxidation resistance at 600–700 °C.

## 1. Introduction

Refractory high entropy alloys (RHEAs) have been designed with high melting point elements based on the concept of high entropy alloys (HEAs), which was first proposed by Senkov et al. in 2010 [1]. Afterward, extensive efforts have been devoted to composition design and property optimization in the RHEA field. Up to now, RHEAs with different alloy systems have been developed, most of which exhibit excellent mechanical properties at high temperatures and significant brittleness at room temperature, while room-temperature (RT) tensile plasticity is generally lower [2,3,4]. The contradiction between strength and plasticity remains the main factor restricting the practical application of RHEAs.

Among the various RHEA systems, TiVNbTa has attracted attention for its combination of excellent high-temperature strength and appreciable room-temperature ductility. Lee et al. [5] reported a NbTaTiV alloy with a room-temperature compressive yield strength of 1273 MPa, retaining 688 MPa at 900 °C with a compressive strain of no less than 30%. Gao et al. [6] prepared a TiVNbTa alloy via mechanical alloying combined with spark plasma sintering, achieving a compressive yield strength of 1506 MPa and a plastic strain of 33%. Scales et al. [7] determined the brittle-to-ductile transition temperature of the as-cast TiVNbTa to be −47 to −27 °C, with an activation energy of approximately 0.52 ± 0.09 eV. Regarding tensile properties, the room-temperature tensile data of pure TiVNbTa were reported by Guo et al. [4] in 2024, with a yield strength of approximately 800 MPa and a fracture elongation of about 40%; whereas the tensile properties of 720 MPa/14% reported by Xu et al. [8] correspond to the hot-rolled TiVNbTaSi_0.1_ alloy. These results indicate that TiVNbTa possesses excellent intrinsic mechanical properties, while the microstructural state of TiVNbTa-based alloys is sensitive to element content and processing conditions. 

Alloying is an important approach to tailoring the microstructure and properties of RHEAs. In TiVNbTa-based alloys, the addition of a third element alters phase stability and mechanical properties. Silicon was added to TiVNbTa RHEA to improve its strength and oxidation resistance by Xu et al. [8]. Aluminum was added to TaNbVTiAl_x_ (x = 0, 0.2, 0.4, 0.6, 0.8, and 1.0) by Li et al. [9], whose study indicated that all these alloys had a single BCC structure and the addition of an appropriate amount of an Al element could improve the compressive strength of TaNbVTiAl_x_ alloys. In addition, variations in Ti content in TiVNbTa-based alloys enable a wide range of strength–ductility combinations [10], and the addition of a small amount of W has endowed as-cast alloys with a favorable strength–ductility combination [11].

As an alloying element with a relatively small atomic radius and distinctive mixing enthalpy characteristics, Cr has attracted considerable attention for its effects on the microstructure and properties of TiVNbTa-based RHEAs. Zhang et al. [12] reported that the addition of Cr to NbTaTiV-(Cr, Zr, W) single-phase coatings introduced the Cr_2_Ta Laves phase and increased the hardness from approximately 373 to 532 HV. Systematic studies on the Cr content in TiVNbTa-based alloys have emerged in recent years. Lv et al. [13] found that in (TiNbVTa)_100-x_Cr_x_ alloys, the addition of Cr (0.35 at.%) simultaneously improved strength and ductility through short-range ordering (SRO) and lattice distortion (YS 903 MPa, elongation 18.7%), while an increase to 0.7 at.% Cr caused grain-boundary segregation and property degradation. Chen et al. [14] investigated TiNbV0.5Ta0.5Cr_x_ (x = 0, 0.1, 0.2, 0.5) alloys and found that the as-cast alloys were single-phase BCC for x ≤ 0.2, while a minor C15 Laves phase appeared at grain boundaries at x = 0.5. Annealing induced precipitation of α-Ti hexagonal close packing (hcp) and C15 Laves phase, with α-Ti preferentially nucleating at grain boundaries, leading to embrittlement. The Laves phase exhibited an incubation period and contributed to strengthening. However, the degradation observed at 0.7 at.% Cr suggested that further increasing the Cr content may not be beneficial. The microstructural evolution and mechanical properties of TiVNbTa-based alloys at substantially higher Cr contents in the as-cast state have not been systematically investigated. In this work, samples with higher Cr content (5.88 and 11.11 at.%) were selected for exploration.

Heat treatment also profoundly influences the microstructural evolution of TiVNbTa RHEAs. Liu et al. [15] examined the effect of heat treatment on the microstructure of Ti-V-Cr5-Nb-Ta and Ti-V-Cr-Nb-Ta alloys. Both alloys showed a near-single-phase BCC structure in the as-cast state. After homogenization at 800 °C, the C15 Laves phase of (Ti, Ta) (V, Cr, Nb)_2_ composition precipitated into the BCC matrix. Homogenization at 1200 °C restored single-phase BCC in the low-Cr alloy, while the Laves phase fraction increased further in the high-Cr alloy. Lv et al. [16] showed that grain refinement and bimodal structure suppress strain localization, significantly improving yield strength (YS) to 1100 MPa and elongation to 25.6% through cold rolling and short-term annealing to the (TiNbVTa)_99.65_Cr_0.35_ alloy. Long et al. [17] investigated the precipitation behavior of TiVNbTaAl_x_ alloys after medium-temperature annealing and its effect on mechanical properties. These studies have largely been confined to specific Cr contents and heat treatment conditions, especially focusing on room-temperature properties. The effects of higher Cr levels and the associated mechanical performance and oxidation behavior at elevated temperatures have not been fully explored. 

High-temperature oxidation resistance is an important aspect of RHEA service performance. Rivers et al. [18] studied the oxidation behavior of TiVCrNbTa and AlTiVCrTa alloys at 1000 °C, finding that substituting Al for Nb significantly reduced the porosity of the oxide scale and improved oxidation resistance. Varma et al. [19] showed through oxidation studies of the Nb-Cr-V-W-Ta alloy at 600–1400 °C that oxide scale cracking and spallation are key factors governing oxidation failure. Bamisaye et al. [20] reported significant mass loss and severe oxide spallation in the TiNbTaVW alloy after 15 h of cyclic oxidation at 850 °C and 1050 °C. Shi et al. [21] also observed the effect of Cr on the oxidation behavior of Ta-Nb-Cr-Ti coatings. Overall, the role of Cr in the oxidation resistance of RHEAs has not reached a consistent conclusion, and the oxidation behavior of TiVNbTa-based alloys at intermediate temperatures (600–700 °C) lacks systematic investigation. 

Table 1 presents the investigation of alloying, processing, microstructural evolution, and mechanical properties of TiVNbTa-based RHEAs. In summary, although the influence of Cr on the microstructure and properties of TiVNbTa- based RHEAs has been preliminarily understood, previous studies have mostly focused on lower Cr content (≤0.7 at.%) or the state after heat treatment. The systematic research on the microstructure evolution of as-cast alloys, mechanical behavior at room temperature and moderate temperature (600 °C), and oxidation behavior at 600–700 °C remains insufficiently studied when the Cr content is higher. In this work, the TiVNbTaCr_x_ (x = 0, 0.25, 0.5) alloys were fabricated by a vacuum suspension induction furnace. The microstructure, tensile properties at room temperature and 600 °C, and oxidation resistance at 600 °C and 700 °C were investigated in detail, and the effects of Cr content on the microstructure evolution, tensile properties and oxidation behavior under high temperature were also discussed, aiming to provide experimental evidence for the alloying design of TiVNbTa-based RHEAs.

## 2. Materials and Methods

TiVNbTaCr_x_ RHEAs cast ingots with x = 0, 0.25 and 0.5, which will be simply denoted by Cr_0_, Cr_0.25_, and Cr_0.5_ hereafter, respectively, were fabricated from 5 kinds of pure metal powders (particle size between 45 and 105μm) with a purity not less than 99.9% in a CXZGX-1 vacuum suspension induction furnace (Jinzhou Heli Co., Ltd., Jinzhou, China). TiVNbTa was purchased from Hebei Jiuyue New Material Technology Co., Ltd (Xingtai, China). Before smelting, the powders were evenly dry-mixed in a KE-0.4L planetary ball mill (Qidong Honghong instrument and equipment factory, Qidong, China) with a rotation rate of 200 r/min for 4 hours. Afterward, approximately 300 g of the alloyed powders were compacted into a cylindrical shape with a diameter of 30 mm using a hollow cylindrical model and a SHW-2000Y extrusion testing machine (Jinan XuLian Instrument Equipment Co., Ltd., Jinan, China) under a force of 50 kN. Subsequently, the smelting process was conducted 12 times to guarantee uniformity of the microstructure and composition. Finally, the alloys were cooled in the water-cooled melting crucible to form button ingots without casting into a model, avoiding oxidation from occurring in the casting procedure.

**Figure 1:**
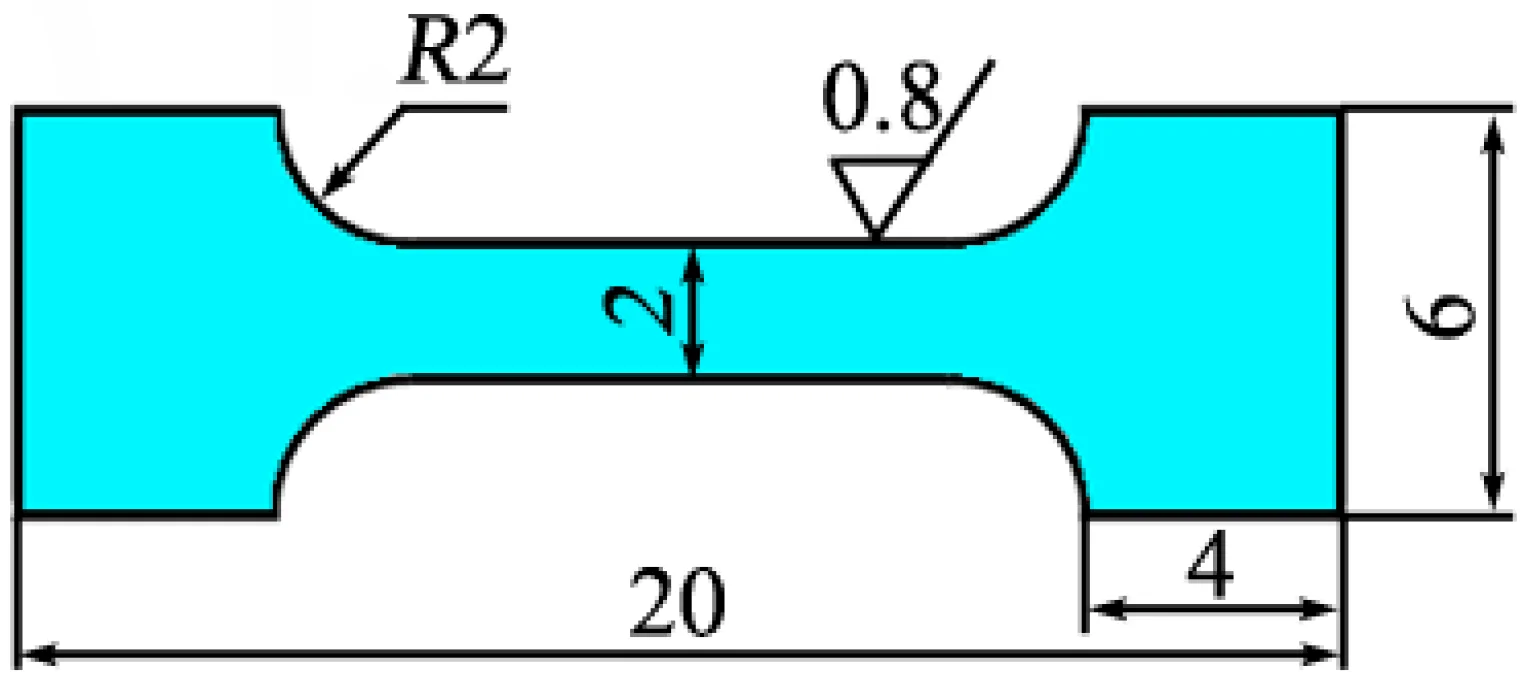
Schematic diagram of the tensile specimens (mm).

In order to study the effect of the Cr element on the mechanical properties of TiVNbTaCr_x_ RHEAs, the tensile specimens were cut using an electric spark cutting machine (TRS-32, Taizhou terui CNC machine Co., Ltd., Taizhou, China) with a width of 6 mm and a length of 20 mm (as shown in Figure 1). The dimensions of the tensile specimens met the national standard GB/T 228.1–2021 [22]. The tensile tests were conducted at a tensile rate of 0.5 mm/min at RT and 600 °C with a LE5105 universal testing machine (Lishi instrument Co., Ltd, Shanghai, China), and at least three specimens were tested for each condition. The measured data were finally calculated as the average value.

The medium-temperature oxidation experiments were performed at 600 °C and 700 °C in a YTH-2.5–12 furnace (Wuhan Huawei kechuang Co., Ltd, Wuhan, China) exposed to the surrounding laboratory air. The samples were cut using electric spark into dimensions of 5 mm × 5 mm × 4 mm. Six separate specimens were heated for 1 h, 5 h, 10 h, 24 h, 35 h, and 50 h at each temperature to study the oxidation resistance of the alloys. The specimens were weighed periodically during oxidation, and the mass change per unit surface area (mg/cm^2^) plotted against oxidation time was adopted to characterize the oxidation resistance of the alloys. These oxidation tests were repeated four times to ensure their reliability.

The microstructure of the TiVNbTaCr_x_ RHEA and the oxidized samples was investigated using an optical microscope (OM) (Axio Imager A2M, ZEISS, Oberkochen, Germany) and scanning electron microscopy (SEM, VEGA3, TESCAN, Brno, Czech Republic) equipped with a back-scattered electron receiver (BSE) and an energy-dispersive spectrometer (EDS, X-act, Oxford Instruments, High Wycombe, UK). The composition of the specimens was measured using an electron probe X-ray micro-analyzer (EPMA, JXA-8530F PLUS, JEOL Ltd., Tokyo, Japan). The phase composition of the TiVNbTaCr_x_ RHEAs and the surface oxidation layers were characterized with an X-ray diffractometer (XRD, SmartLab 9X, Rigaku Corporation, Tokyo, Japan) at a scanning rate of 1°/min. A monochromatic Cu-kα (λ = 0.15402 nm) was used as a target material with a scan range of 20°–100°. The lattice strain of TiVNbTaCr_x_ RHEAs was obtained through a Williamson–Hall (W–H) plot [23]. The fine microstructure features and phase information of the RHEA were revealed through a transmission electron microscope (TEM, Talos F200X, FEI Company, Brno, Czech Republic). 

## 3. Results

### 3.1. Phase Composition and Microstructure Analysis

**Figure 2:**
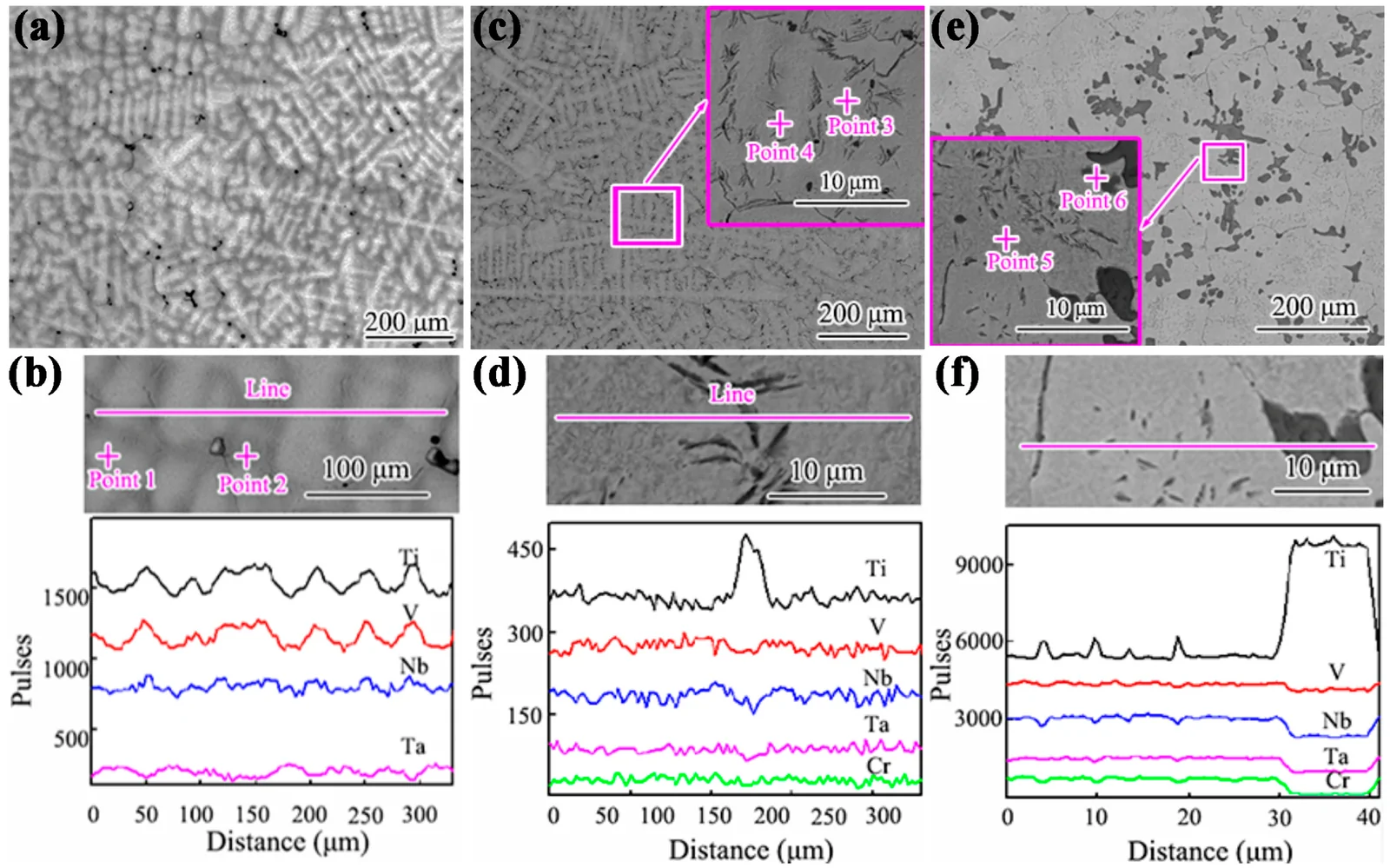
BSE image and EDS scanning results: (**a**,**b**) Cr_0_, (**c**,**d**) Cr_0.25_, and (**e**,**f**) Cr_0.5_.

Figure 2 shows the BSE images and EDS scanning results of Cr_0_, Cr_0.25_, and Cr_0.5_ RHEAs, respectively. The dendritic structures are clearly visible in the BSE morphology (Figure 2a,c,e). The point compositions of the dendritic and interdendritic regions in Cr_0_, Cr_0.25_, and Cr_0.5_ alloys are shown in Table 2. Compared with the interdendritic regions, the dendritic areas show a distinctly brighter contrast under observation, which indicates that elements with relatively high atomic numbers are enriched in the dendritic phase, while the interdendritic regions present the opposite elemental distribution feature.

The EDS was conducted, and compositions of the dendritic and interdendritic regions are shown in Figure 2b,d,e. The EDS results indicated that the bright white dendritic region is rich in Ta, while the gray interdendritic region is rich in Ti (Figure 2b). Different from Cr_0_, the Cr_0.25_ alloy has a straw-like phase formed in the interdendritic areas, which is enlarged in Figure 2c, and the EDS line-scanning (Figure 2d) and point-scanning (Table 2) results indicated that the straw-like phase was rich in Ti to a level of about 60 at.%. Simultaneously, the atomic percentage of Cr in the matrix phase is about 5.37%. The Cr_0.5_ alloy mainly contains three kinds of microstructures, and they were a bright white matrix, a short rod-shaped Ti-rich phase (Similar to the straw-like Ti-rich phase in the Cr_0.25_ alloy), and a dark gray block-like phase (Figure 2e). EDS line-scanning (Figure 2f) and point-scanning (Table 2) indicated that the atomic percentage of Cr in the matrix phase increased to about 11.25%, and the dark gray block-like phase contains a Ti element with an atomic percentage of approximately 82.14%. The microscale compositional evolution of TiVNbTa alloy induced by Cr addition is fundamentally attributed to the preferential crystallization of high-melting-point elements during solidification [14].

**Figure 3:**
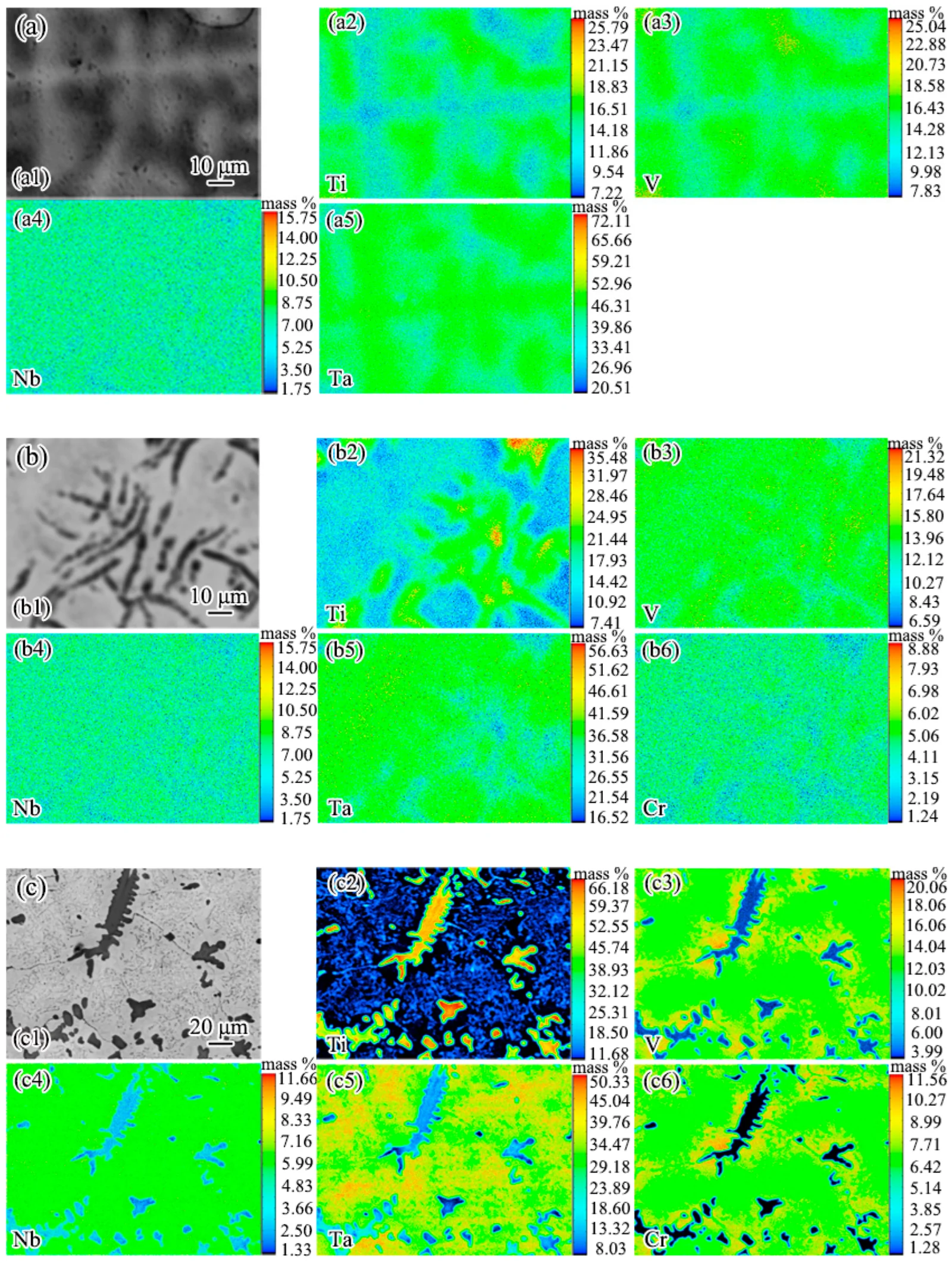
EPMA maps of TiVNbTaCr_x_: (**a**) Cr_0_, (**b**) Cr_0.25_, and (**c**) Cr_0.5_.

In order to observe the elemental distribution of the three alloys more intuitively and verify the above morphology and composition analysis, the EPMA surface scanning of these alloys was carried out, and the results are shown in Figure 3. The EPMA map in Figure 3a verified the dendritic structure of Cr_0_ alloy. In the Cr_0_ alloy, the high-melting-point elements are enriched in the dendritic regions, such as Ta with a melting point of 3014 °C (Figure 3(a5)), whereas the lower-melting-point elements (Ti, V) gather in the interdendritic regions (Figure 3(a2,a3)). Meanwhile, it also shows that the distribution of Nb is more uniform than other elements, which is consistent with the findings presented study [8,13,15]. The EPMA maps of Cr_0.25_ and Cr_0.5_ (Figure 3b,c) also reflect the significant enrichment of the Ta element in the dendritic region, while the enrichment of the Ti element in the interdendritic region becomes more severe with the addition of the Cr element. Overall, Ti segregation has been exacerbated by the addition of the Cr element in the TiVNbTa alloy, and the higher the Cr content, the more severe the Ti segregation.

**Figure 4:**
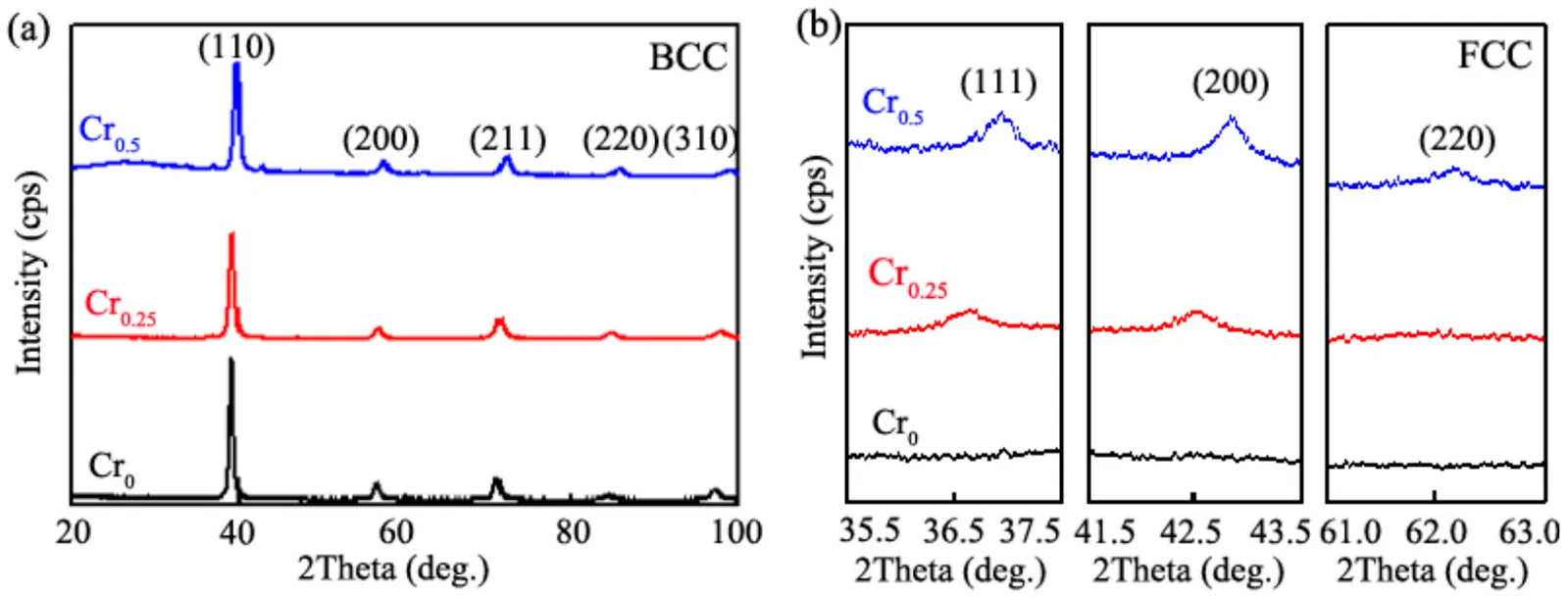
XRD pattern of TiVNbTaCr_x_ alloys: (**a**) XRD pattern, (**b**) enlarger view.

The XRD pattern of Cr_0_, Cr_0.25_, and Cr_0.5_ alloys is shown in Figure 4a, and the detailed scanning of three areas is displayed in Figure 4b. Combining with the above microstructure and composition analysis results, Cr_0_ alloy has a single body-centered cubic (BCC) structure, which is consistent with the existing literature reports [7,8,24]. The detailed scanning results in Figure 4b show that a small amount of face-centered cubic (FCC) phase formed with the addition of Cr. It can be inferred from the microstructure and composition analysis in Figure 2 that the FCC phase mainly contains the Ti element, as well as a small amount of Nb, Ta, and V. 

It can also be analyzed from the XRD results that Cr addition influences the lattice strain and lattice constant of the dominant BCC phase. The existence of the BCC phase was determined by the diffraction peaks, i.e., (110), (200), (211), (220), and (310), as shown in Figure 4a, which are very close to those of TaNbV (PDF Card NO.: 65–4825). According to the Williamson–Hall (W–H) plot analysis results, the lattice strain of Cr_0_, Cr_0.25_, and Cr_0.5_ alloys are 2.59 × 10^−3^, 3.34 × 10^−3^, and 1.71 × 10^−3^, respectively. It indicates that as the Cr atomic percent increases from 0 to 11.25%, the lattice strain of the BCC phase first increases and then decreases. The interplanar spacing of the BCC phase in the three RHEAs was calculated using Bragg’s law, and their lattice constants were further calculated based on the results. The results indicate that the BCC phase lattice constants of Cr_0_, Cr_0.25_, and Cr_0.5_ alloys are 3.2496 Å, 3.2411 Å, and 3.2050 Å, respectively. This is because the atomic radius of Cr (1.25 Å) is smaller than that of Ti (1.47 Å), V (1.32 Å), Nb (1.43 Å), and Ta (1.43 Å), so its integration leads to a slight decrease in the lattice constant.

**Figure 5:**
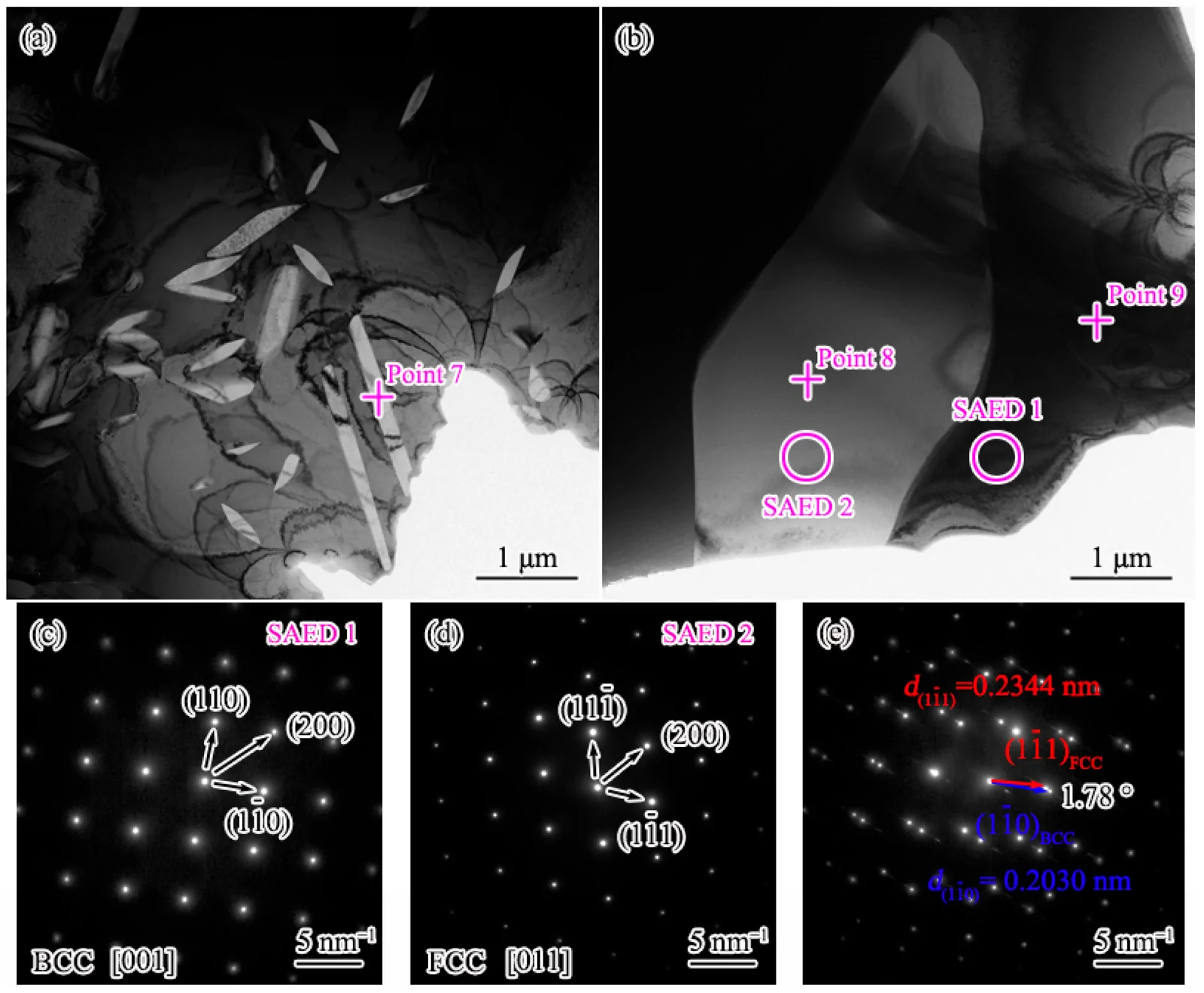
TEM analysis of Cr_0.5_: (**a**,**b**) Bright-field image, (**c**–**e**) SAED of the BCC matrix and FCC Ti-rich phase.

To further confirm the composition and phase analyses, TEM testing was performed on the Cr_0.5_ alloy (Figure 5), and the compositions of points were listed in Table 3, which contains both short rod-like and blocky Ti-rich precipitates with distinct morphologies and the highest Ti content (~82 at.%), so as to unambiguously determine the crystal structure and composition of the precipitated phases. The bright-field images are shown in Figure 5a,b, in which the black contrast area was the matrix of the Cr_0.5_ alloy, while the white bright contrast area was the Ti-rich precipitates. The compositions of the matrix and Ti-rich phase were examined using TEM-EDS, and the results are shown in Figure 5(b1), which were consistent with the previous composition analysis in Figure 2. The selected area electron diffraction (SAED) of the matrix (SAED 1) and Ti-rich precipitation (SAED 2) is shown in Figure 5c–e, respectively. The results showed that the crystal structures of the matrix and the Ti-rich phase are BCC and FCC, respectively, which are completely consistent with the XRD analysis results in Figure 4. 

In order to explore the reasons for the precipitation of the Ti-rich phase, the important empirical parameters containing mixing entropy (Δ*S*_mix_, Equation (1)), mixing enthalpy (Δ*H*_mix_, Equation (2)), thermodynamic parameter (*Ω*, Equation (3)), atomic size difference (*δ*_r_, Equation (4)), valence electron concentration (*VEC*, Equation (5)), and atomic packing parameter (*γ*, Equation (6)) have been calculated. Meanwhile, the density of the alloys has also been estimated by the rule of mixtures (*ρ*, Equation (7)).

(1) $$∆S_{mix}=-R\sum_{i=1}^{n}c_{i}lnc_{i}$$

The *c_i_* and *c_j_* represent the atomic percentages of the *i*th and *j*th elements hereafter.

(2) $$∆H_{mix}=\sum_{i=1,i\neq j}^{n}4∆H_{ij}^{mix}c_{i}c_{j}$$

where $∆H_{ij}^{mix}$ is the mixing enthalpy of a binary liquid alloy composed of the *i*th and *j*th principal elements in regular solution.

(3) $$\Omega =\frac{T_{m}{∆S}_{mix}}{\left|{∆H}_{mix}\right|}$$

where $T_{m}=\sum_{i=1}^{n}c_{i}{{(T}_{m})}_{i}$ and ${{(T}_{m})}_{i}$ is the melting temperature of the *i*th elements with Kelvin units.

(4) $$\delta_{r}=\sqrt{\sum_{i=1}^{n}c_{i}{\left(1-\frac{r_{i}}{\bar{r}}\right)}^{2}}$$

where $\bar{r}=\sum_{i=1}^{n}c_{i}r_{i}$, and the $r_{i}$ is the atomic radius of the *i*th elements.

(5) $$VEC=\sum_{i=1}^{n}c_{i}{\left(VEC\right)}_{i}$$

where ${\left(VEC\right)}_{i}$ represents the valence electron concentration of the *i*th elements.

(6) $$\gamma =\frac{1-\sqrt{\left[{\left(r_{s}+\bar{r}\right)}^{2}-{\bar{r}}^{2}\right]/{\left(r_{s}+\bar{r}\right)}^{2}}}{1-\sqrt{\left[{\left(r_{L}+\bar{r}\right)}^{2}-{\bar{r}}^{2}\right]/{\left(r_{L}+\bar{r}\right)}^{2}}}$$

where $r_{s}$ and $r_{L}$ are the smallest and the largest atomic radii among the constituent elements of the alloy, respectively.

(7) $$\rho =\sum A_{i}c_{i}/\sum \frac{A_{i}c_{i}}{\rho_{i}}$$

where the *A_i_* and *ρ_i_* represent the relative atomic mass and density of the *i*th elements, respectively.

During the calculation process, the atomic radius, melting point, density, and relative atomic mass parameters of Ti, V, Nb, Ta, and Cr elements were selected from the literature [25]. $∆H_{ij}^{mix}$ and ${\left(VEC\right)}_{i}$ were taken from the literature [26] and [27], respectively.

The calculation results are shown in Table 4. According to the solid solution phase formation rules ($\delta_{r}\leq 6.5\%$, $-15\leq ∆H_{mix}\leq 5$ kJ/mol, and $12\leq ∆S_{mix}\leq 17.5$ J K^−1^ mol^−1^) proposed by Zhang et al. [28], Cr_0.25_ and Cr_0.5_ alloys should form a solid solution phase. Further considering the later solid solution phase formation rules of Ω≥1 and $\delta_{r}\leq 6.6\%$ proposed by Yang et al. [29] and VEC<6.87 proposed by Guo et al. [27], the predicted result is still that sole BCC phase would be generated in Cr_0.25_ and Cr_0.5_ alloys. According to the atomic packing criterion γ<1.175 revealed by Wang et al. [30], Cr_0.25_ and Cr_0.5_ alloys would possibly lose the BCC single-phase solid solution due to the instability of atomic stacking caused by the large difference in atomic size. It can be inferred that the precipitation of Ti after the addition of Cr may be associated with the reduced stability of the BCC single-phase solid solution, as reflected by the *γ* criterion and the intensified Ti segregation caused by Cr addition.

### 3.2. Mechanical Properties

**Figure 6:**
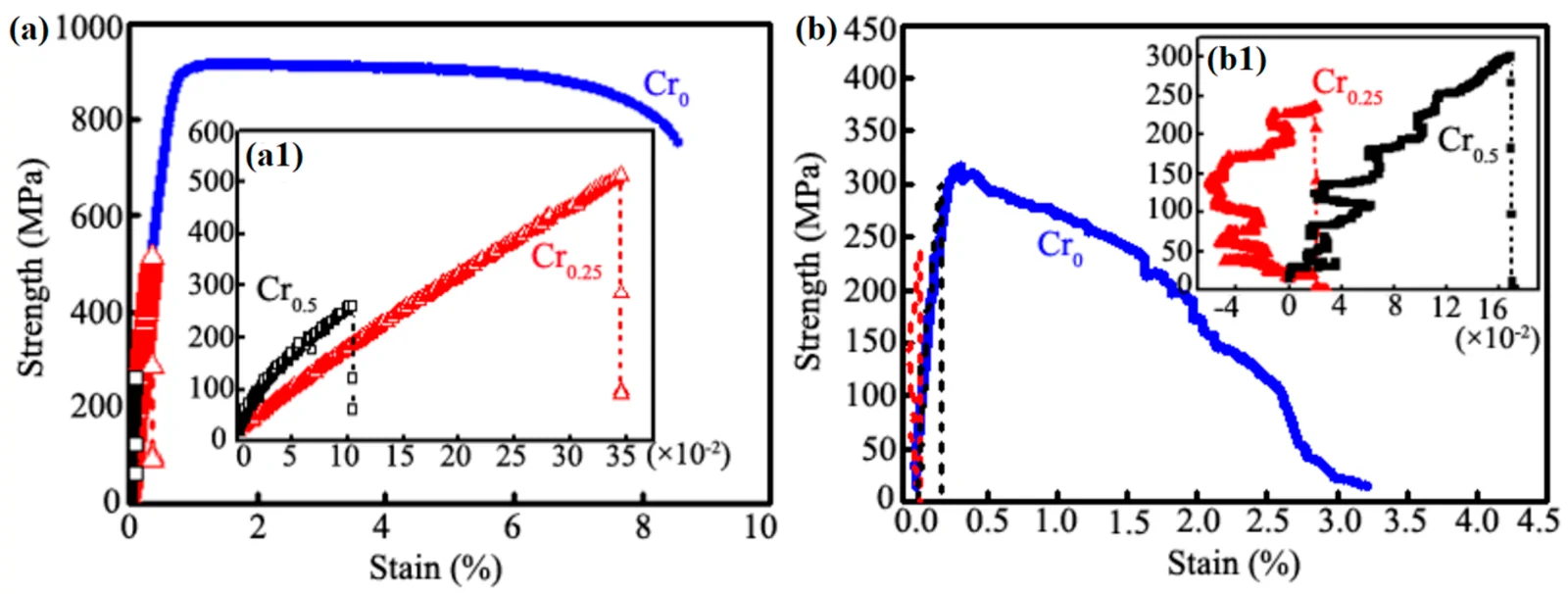
Tensile stress–strain curves of TiVNbTaCr_x_ alloy at room temperature and 600 °C: (**a**) Room temperature, (**b**) 600 °C.

Figure 6 presents the tensile stress–strain curves for TiVNbTaCr_x_ alloy (x = 0, 0.25, 0.5) at RT and 600 °C. The details are enlarged in Figure 6(a1,b1), the results of the tensile tests are listed in Table 5. The Cr0 alloy displays a tensile strength of 915 MPa with an elongation of 8.56% at RT. With the addition of Cr, the tensile strength of Cr_0.25_ and Cr_0.5_ alloys substantially decreases to 513 MPa and 261 MPa, while the ductility degrades to ~0.35% and ~0.1%, respectively. Brittle fracture occurred for the Cr_0.25_ and Cr_0.5_ alloys. Generally, with the increase in the Cr element content, the room-temperature tensile strength and plasticity of TiVNbTaCr_x_ (x = 0, 0.25, and 0.5) alloys rapidly decrease. At 600 °C, the Cr_0_ alloy also has higher tensile strength (316 MPa) and elongation (3.22%) than the other two alloys. Compared with those at RT, the strength of the Cr_0.25_ alloy decreases by about 50%, while its plasticity is almost lost. The strength and plasticity of the Cr_0.5_ alloy do not change significantly at 600 °C and room temperature, which may be due to the fact that the influence of temperature is weaker than that of the brittle structure itself.

**Figure 7:**
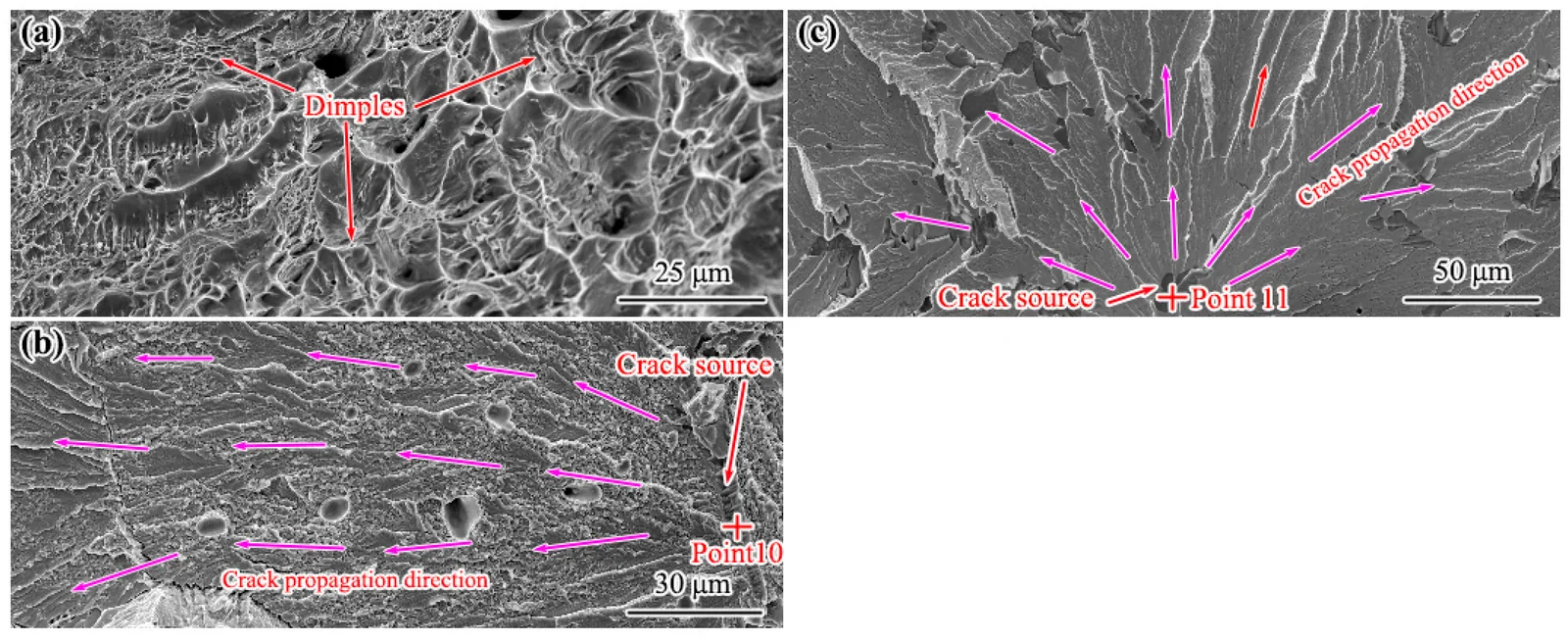
Fracture characteristics and performance: (**a**) Cr_0_ at RT, (**b**) Cr_0.25_ at RT, (**c**) Cr_0.5_ at RT.

The fracture morphology, composition, and tensile properties are depicted in Figure 7. Figure 7a–c show the tensile fracture morphology of Cr_0_, Cr_0.25_, and Cr_0.5_ alloys, respectively. The fracture surface of the Cr_0_ alloy shows dimple morphology (Figure 7a), indicating ductile fracture, while the fracture surfaces of Cr_0.25_ (Figure 7b) and Cr_0.5_ (Figure 7c) show cleavage fracture characteristics, suggesting brittle fracture. In Figure 7b,c, the crack source was indicated in reverse along the radial pattern shown by the pink arrows, whose composition was determined by EDS point scanning in Table 6. The results suggested that the cracks in Cr_0.25_ and Cr_0.5_ alloys originated from the needle-like or block-like Ti-rich phase during the stretching process. These results indicated that regardless of the test temperature, the Cr_0_ alloy exhibited a certain degree of ductile fracture characteristics, while the other two alloys exhibited brittle fracture characteristics, which can be inferred from the tensile curves in Figure 6a,b and the fracture morphology in Figure 7a–c.

Combining the results of microstructure and tensile performance analysis, it can be inferred that with the addition of Cr, the single-phase structure of BCC was fragmented by needle-like and block-like Ti-rich phases, which led to stress concentration during the tensile process, resulting in a decrease in strength and ductility.

### 3.3. Oxidation Behavior Under High Temperature

**Figure 8:**
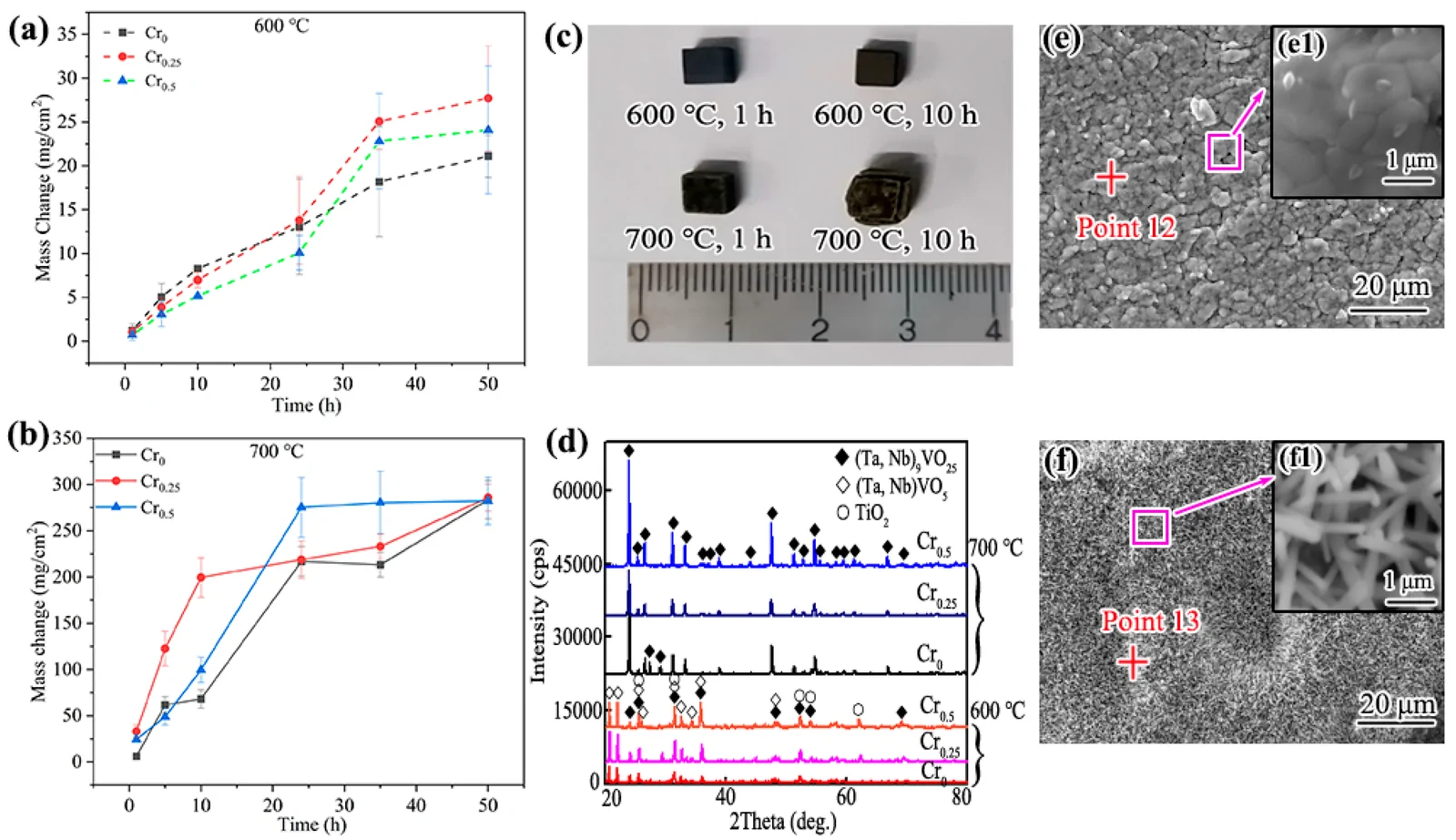
Oxidation resistance and the oxidation layer: (**a**,**b**) Oxidation kinetics curves at 600 °C and 700 °C, (**c**) Macroscopic morphology, (**d**) XRD pattern of the oxidation layer, (**e**,**f**) Oxidation layer morphology at the early stage and the mature stage of Cr_0.5_ oxidized at 600 °C.

Figure 8a shows the oxidation kinetics curves of Cr_0_, Cr_0.25_, and Cr_0.5_ at 600 °C in an air atmosphere. When the oxidation time did not exceed 10 h, the mass gain rate of the Cr_0.5_ alloy was slightly lower than those of the Cr_0_ and Cr_0.25_ alloys, the latter two being similar, indicating that the addition of an appropriate amount of Cr suppressed the mass gain rate to a certain extent during the initial oxidation stage. With further extension of the oxidation time beyond 35 h, the mass gain of the Cr_0_ alloy tended to level off, which can be attributed to the protection of the underlying metal by a dense oxide film formed on the surface. In contrast, the mass changes of the Cr_0.25_ and Cr_0.5_ alloys continued to increase, possibly because the addition of Cr reduced the integrity of the oxide film, making it prone to cracking and spallation from the substrate. The same phenomenon also occurred after holding at 700 °C for more than 10 h (Figure 8c).

Figure 8b shows the oxidation kinetics curves of the three alloys oxidized in air at 700 °C. At this temperature, the mass gains of all three alloys were considerably higher than those at 600 °C, indicating that the increase in temperature markedly intensified the oxidation. In contrast to the behavior at 600 °C, the differences in mass gain among the three alloys at 700 °C gradually diminished with increasing oxidation time and approached similar values after 50 h, suggesting that the influence of Cr content on the total mass gain tended to weaken at higher temperatures.

Due to the surface oxidation layer peeling off after being held for 50 h at 600 °C and cracking significantly after being held for 10 h at 700 °C, it was meaningless to further extend the holding times. Figure 8c displays the typical macrostructure morphology of the Cr_0.5_ alloys oxidized at 600 °C and 700 °C for 10 h and 1 h, respectively. In summary, the addition of Cr exerted a slight suppressing effect on the mass gain rate of the TiVNbTaCr_x_ alloys during the initial oxidation stage, but it did not hinder the continued mass gain during the long-term oxidation process. Raising the temperature from 600 °C to 700 °C significantly intensified the oxidation, while the differences in oxidation behavior among the three alloys diminished with increasing temperature.

Figure 8d shows the XRD patterns of the surface oxidation layer obtained at 600 °C and 700 °C of Cr_0.5_. Figure 8e,f show the surface oxidation layer morphology at the early stage and mature stage, respectively. The EDS testing results of points 12–13 are listed in Table 7. The growth process of the oxide film of the three alloys was the same. The surface oxidation layer mainly contains Ta, Nb, and V elements, which may be (Ta, Nb)_9_VO_25_ oxide, possessing a small amount of Ti atoms. There may be some TiO_2_ and (Ta, Nb)VO_5_ oxide formed at 600 °C. It can also be found that there was a very limited amount of Cr in the surface oxidation layer, let alone the formation of chromium oxides. As oxidation continued, the oxide film gradually grew from thin film shape (Figure 8e) into a fibrous shape (Figure 8f).

**Figure 9:**
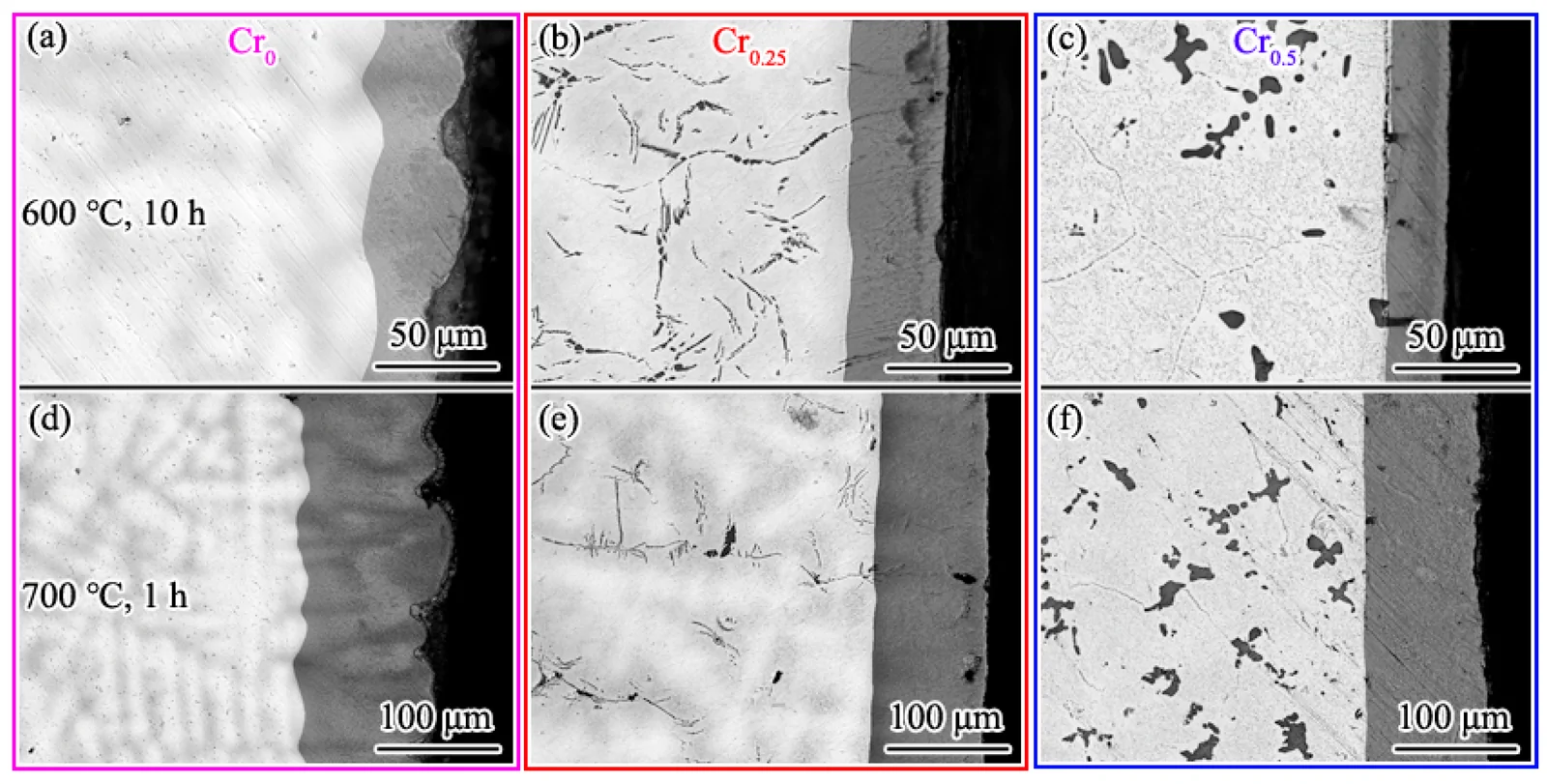
Surface oxidation layer of TiVNbTaCrx: (**a**–**c**) Cr_0_, Cr_0.25_, and Cr_0.5_ at 600 °C/10 h, (**d**–**f**) Cr_0_, Cr_0.25_, and Cr_0.5_ at 700 °C/1 h.

The cross-sections of the samples achieved at 600 °C for 10 h and 700 °C for 1 h were observed, as shown in Figure 9. From the cross-section morphology, it can be found that the surface oxide layers of the three alloys were all dense under different oxidation conditions. Moreover, under the same oxidation condition, the surface oxidation layer thicknesses of the three alloys were similar. Based on the experimental results, within the composition range investigated (x ≤ 0.5), no chromium oxide film was observed on the alloy surface after oxidation, and the addition of Cr did not improve the medium-temperature (600–700 °C) oxidation resistance of the TiVNbTaCr_x_ alloys in this study.

## 4. Discussion

### 4.1. Cr-Triggered FCC Ti-Rich Precipitation and Microstructure Evolution

TiVNbTa-based RHEAs typically solidify as a single BCC phase, yet dendritic segregation is nearly unavoidable due to differences in elemental melting points and solute redistribution. Senkov et al. [1] and Couzinié et al. [31] reported Ta/Nb enrichment in dendrites and Ti/V segregation into interdendritic regions in NbMoTaW and TiZrHfNbTa alloys, respectively. The BCC dendritic structure of the Cr_0_ alloy and its EPMA results are consistent with these reports, with Nb displaying the most uniform distribution as noted by Refs [8,13,15]. The Cr_0_ alloy also satisfies the solid–solution formation criteria of Guo et al. [27], Zhang et al. [28] and Yang [29]. The addition of Cr fundamentally alters the solidification pathway. A straw-like FCC Ti-rich phase (~60 at.% Ti) appears in the interdendritic regions of the Cr_0.25_ alloy (Cr 5.88 at.%). At 11.11 at.% Cr (Cr_0.5_), the Ti-rich phase evolves into short rod-like and blocky morphologies (~82 at.% Ti), identified by XRD and TEM/SAED. EPMA confirms that Cr intensifies Ti segregation. As the Cr content increases from 5.88 to 11.11 at.%, the Ti-rich phase exhibits a gradual transition from a straw-like to a blocky morphology, accompanied by significant increases in both volume fraction and spatial connectivity. 

Within a comparable Cr range (~5-12 at.%), different precipitation pathways have been reported: Chen et al. [14] observed α-Ti and Laves in TiNbV_0.5_Ta_0.5_Cr_x_ (x = 0, 0.1, 0.2, 0.5) after annealing; Liu et al. [15] found C15 Laves in Ti-V-Cr5-Nb-Ta only after homogenization at 800 °C; while Lv et al. [13,16] maintained single-phase BCC at Cr ≤ 0.7 at.%. These comparisons demonstrate that the Cr-induced precipitation pathway is highly matrix-dependent. Cr preferentially promotes an FCC Ti-rich phase in the equimolar TiVNbTa matrix in this study. Cr and Ti exhibit the most negative mixing enthalpy (−7 kJ/mol) [26], providing the thermodynamic driving force. During non-equilibrium solidification, the preferential crystallization of high-melting-point elements (Ta) enriches lower-melting-point elements (Ti, V, Cr) in the residual interdendritic liquid; since the equilibrium partition coefficient of Cr is less than unity, Cr further partitions into the interdendritic liquid, intensifying local solute enrichment [13] and raising the Ti supersaturation beyond the solubility limit, thereby triggering the precipitation of the FCC Ti-rich phase. Therefore, in both Cr-containing alloys investigated (5.88 and 11.11 at.%), the Ti supersaturation in the interdendritic regions is sufficient to trigger the precipitation of the FCC Ti-rich phase.

The lattice distortion criterion of Toda-Caraballo et al. [32] further describes the relationship between atomic size mismatch and phase stability in HEAs. The atomic packing parameter *γ* > 1.175 (Table 4) proposed by Wang et al. [30] successfully predicts the destabilization of the BCC single-phase structure, whereas conventional parameters fail to capture this transition. The monotonic decrease in lattice constant (3.2496 → 3.2050 Å) and the non-monotonic lattice strain (2.59 → 3.34 → 1.71 × 10^−3^) corroborate the compositional reorganization of the BCC matrix following Ti-rich phase precipitation.

### 4.2. Effect on Mechanical Behavior

Cr addition induces catastrophic deterioration of room-temperature tensile properties. The Cr_0_ alloy exhibits a tensile strength of 915 MPa and an elongation of 8.56% with dimple fracture (Figure 7a). At 5.88 at.% Cr (Cr_0.25_), the strength drops to 513 MPa and elongation to 0.35%, with cleavage fracture (Figure 7b). At 11.11 at.% Cr (Cr_0.5_), the strength falls to 261 MPa and elongation to 0.10% (Figure 7c). This degradation far exceeds that at microalloying levels (Cr ≤ 0.7 at.%): Lv et al. [13] reported only 8% strength loss in (TiNbVTa)_99.3_Cr_0.7_; Chen et al. [14] maintained single-phase BCC with good ductility in as-cast Cr_0.2_ (~6.25 at.%). Backtracking radial patterns on fracture surfaces with SEM and EDS results (Figure 7b and Table 6) confirm that crack initiation occurs within the FCC Ti-rich phase and at the FCC/BCC interface. The Ti-rich phase is usually located in the interdendritic regions and grain boundaries due to the slow diffusion of Ti atoms. It can be inferred that the embrittlement pathway is similar to the crack propagation caused by α-Ti grain boundary precipitation [14], but different from grain boundary Cr segregation [13] and BCC/Laves phase interfacial embrittlement [15]. The atomic packing parameter *γ* > 1.175 proposed by Wang et al. [30] predicts atomic-scale packing instability within the FCC phase, which may serve as the structural origin of its intrinsic brittleness, consistent with the Ti-rich composition (~82 at.% Ti, Table 6) of the crack-initiating phase.

This study provides the mechanical properties at 600 °C (Figure 6b). Compared with room temperature, the Cr_0_ alloy retains a degree of ductile fracture at 600 °C (elongation of 3.22%), whereas all Cr-containing alloys exhibit brittle fracture at both room temperature and 600 °C (elongation < 0.20%). This indicates that the FCC Ti-rich phase continues to act as the dominant factor governing the embrittlement of the Cr-containing alloys within the tested temperature range. Microstructurally, the FCC Ti-rich phase precipitates predominantly in the interdendritic regions and along grain boundaries, disrupting the BCC matrix continuity and weakening grain boundary cohesion. Such microstructural degradation facilitates crack initiation at grain boundaries, accounting for the marked reduction in ductility. In summary, regardless of temperature, the precipitation of FCC-rich Ti phase has reduced the ductility of Cr-containing alloys to near-zero levels, and its impact on deformation ability may exceed the strength fluctuations caused by temperature-dependent changes in the BCC matrix.

### 4.3. Effect on High-Temperature Oxidation Behavior

The oxidation kinetics curves (Figure 8a,b) show that at 600 °C, the Cr_0.5_ alloy exhibits a slightly lower initial mass gain rate than the Cr_0_ and Cr_0.25_ alloys, yet the Cr_0_ alloy enters a plateau after 35 h while both Cr-containing alloys continue to gain mass. At 700 °C, the mass gains of all three alloys increase markedly, and the differences among them diminish with time. This behavior indicates that Cr addition exerts only a marginal suppressing effect on the mass-gain rate during the initial oxidation stage, whereas it accelerates mass gain during long-term oxidation due to the reduced integrity of the oxide film. This is consistent with the dual role of Cr in oxidation behavior reported by Zhang et al. [33], where an appropriate Cr content promotes a protective oxide layer while excessive Cr weakens the protection. At 700 °C, oxide spallation occurs more rapidly (~10 h). Varma et al. [19] reported analogous two-stage oxidation behavior and oxide-scale cracking in Nb-Cr-V-W-Ta alloys, suggesting that the scale-stability issues arising from competitive multi-element oxidation in BCC RHEAs warrant attention.

The combination of EDS results (Table 7) and XRD results (Figure 8d) shows that the oxide scale is primarily enriched in Ta, Nb, and V, identified as (Ta,Nb)_9_VO_25_ with minor TiO_2_ and (Ta,Nb)VO_5_, while Cr is virtually absent. Despite the matrix Cr content of 5.88–11.11 at.%, oxygen preferentially reacts with Nb, Ta, and V at 600–700 °C, and Cr_2_O_3_ does not become the dominant phase in the oxide scale. This contrasts with Cui et al. [34], who formed protective oxides via Cr and Al in a different RHEA. Rivers et al. [18] similarly observed Nb/Ta-dominated oxides in Ti-V-Cr-Nb-Ta and reported that Al substitution for Nb significantly improved oxidation resistance. The oxide spallation may be related to microstructural heterogeneity from the coexistence of BCC and FCC Ti-rich phases. TEM (Figure 5) shows that the FCC phase regions are Ti-rich (~82 at.%), while the BCC matrix regions are enriched in Nb and Ta. The compositional differences between regions may lead to spatial variations in oxide growth rate and stress state, thereby affecting the integrity of the oxide scale. Varma et al. [19] also attributed the cracking of oxide scales to the mismatch in thermal expansion coefficients between complex oxides and the substrate. This mechanism is conceptually analogous to the interfacial stress at BCC/C15 phase boundaries reported by Liu et al. [15]. In summary, under the present experimental conditions, no protective Cr_2_O_3_ scale is observed, and the addition of Cr does not improve the long-term oxidation resistance of the TiVNbTaCr_x_ alloys at 600–700 °C. For Nb-, Ta-, and V-rich RHEAs, alternative alloying elements such as Al or Si should be considered [18,33].

## 5. Conclusions

In this work, the effects of Cr addition on the microstructure, mechanical properties, and oxidation resistance of TiVNbTaCr_x_ RHEA (x = 0, 0.25, 0.5) were investigated. The main conclusions are drawn as follows:

(1) The TiVNbTaCr_x_ RHEA (x = 0) exhibits a dendritic structure. The addition of Cr alters the solidification pathway of TiVNbTa alloys from a single-phase BCC solid solution to a BCC matrix with FCC Ti-rich precipitates. With Cr addition (x = 0.25, 0.5), the Ti-rich phases precipitate. The volume fraction of this phase increases with Cr content, and its morphology evolves from straw-like to block-like.
(2) The TiVNbTa alloy possesses favorable strength and ductility at room temperature (915 MPa/8.56%) and 600 °C (316 MPa/3.22%). The addition of Cr markedly degrades these properties, with the room-temperature tensile strength and elongation decreasing by 44% and 96% for the Cr_0.25_ alloy and by 71% and 99% for the Cr_0.5_ alloy, and with extremely low elongations at 600 °C.
(3) The FCC Ti-rich phase precipitates predominantly in the interdendritic regions and along grain boundaries, disrupting the continuity of the BCC matrix and weakening grain boundary cohesion, which promotes crack initiation at grain boundaries and leads to a transition from ductile to brittle fracture in the Cr-containing alloys.
(4) During oxidation tests at 600–700 °C, Cr addition provides only a marginal beneficial effect during the initial stage (0–10 h at 600 °C), while the Cr-containing alloys continue to gain mass upon prolonged exposure due to the reduced integrity of the oxide film. The oxide scale consists primarily of (Ta,Nb)_9_VO_25_, with virtually no Cr detected, and no protective Cr_2_O_3_ scale is observed under the present experimental conditions.

## Figures and Tables

**Table 1:** Summary of alloying, processing, microstructural evolution, and mechanical properties of TiVNbTa-based RHEAs.

Alloy (Cr, at.%)	Investigated Content	Findings	Ref.
TiVNbTa (0)	As-cast; room temperature (RT) uniaxial tensile tests	RT tensile yield strength of ~800 MPa/fracture elongation ~40%	[4]
TiVNbTa (0)	Homogenization; RT to 900 °C compressive tests with in situ neutron diffraction	RT compressive yield strength (YS) 1273 MPa; 688 MPa at 900 °C; compressive strain ≥30%	[5]
TiVNbTa (0)	Mechanical alloying combined with spark plasma sintering; sintering temperature (900–1300 °C) and oxygen/nitrogen contents as variables	Compressive yield strength of 1506 MPa; plastic strain 33% (sintered at 1100 °C)	[6]
TiVNbTa (0)	Four-point bending tests at multiple temperatures; fracture toughness and activation energy	Brittle-to-ductile transition temperature of −47 to −27 °C; activation energy ~0.52 eV	[7]
TiVNbTa (0)	Equimolar TiVNbTa; tensile tests	Tensile yield strength ~720 MPa/elongation ~14%	[8]
TiVNbTaSi_0.1_ (0)	Silicon alloying; as-cast followed by hot rolling; tensile tests and oxidation evaluation	Tensile yield strength of 1250 MPa/elongation 8% after hot rolling	[8]
(TiNbVTa)_100-x_C_x_ (0.35, 0.7)	Cr content gradient (0–0.7 at.%); as-cast; RT tensile tests	0.35 at.%: YS 903 MPa/elongation 18.7% (optimum); 0.7 at.%: Cr segregation at grain boundaries, property degradation	[13]
TiNbV_0.5_Ta_0.5_Cr_x_ (3.23–12.5)	Cr content gradient (0–12.5 at.%); as-cast and annealed (600–1000 °C, 6 h); RT tensile tests	x = 0.1 optimum: YS 878 MPa/elongation 21.6%; α-Ti (hcp) and C15 Laves phase precipitation after annealing	[14]
Ti-V-Cr-Nb-Ta (5, 20)	Cr content (5 vs 20 at.%); as-cast and homogenized (800/1200 °C, 48 h); nanoindentation and microstructural characterization	C15 Laves phase (hardness ~14 GPa); severe embrittlement at 20 at.% Cr	[15]
Ti-V-Cr-Nb-Ta (5, 20)	Oxidation at 1000 °C in air; Nb/Al substitution as variable; Thermogravimetric analysis and oxide scale characterization	Complex oxide scales; Al substitution reduces porosity and improves oxidation resistance	[18]
TiNbTaVW (0)	Cyclic oxidation at 850 °C and 1050 °C (15 h); oxide scale characterization	Significant mass loss and severe oxide spallation	[20]

**Table 2:** Compositions of points in the dendritic and interdendritic regions (at.%).

Point	Ti	V	Nb	Ta	Cr
1	24.13	19.36	26.11	30.40	-
2	29.68	23.96	25.04	21.32	-
3	24.37	23.15	25.60	21.51	5.37
4	63.62	10.26	15.55	10.24	0.33
5	20.13	21.17	25.19	22.26	11.25
6	82.14	3.05	7.89	6.06	0.86

**Table 3:** Compositions of points in the Cr_0.5_ alloy (at.%).

Point	Ti	V	Nb	Ta	Cr
7	86.45	3.31	6.34	3.42	0.48
8	86.76	4.71	5.41	2.58	0.54
9	11.82	22.74	28.55	25.87	11.02

**Table 4:** The calculation results of three alloys.

Alloy	*ρ*(g/cm^3^)	*T*_m_(K)	Δ*H*_mix_(KJ/mol)	Δ*S*_mix_(J K^−1^ mol^−1^)	*Ω*	*δ*_r_(%)	*VEC*	*γ*
Cr_0_	9.15	2540	−0.25	11.56	117.4	4.0	4.75	1.122
Cr_0.25_	9.07	2516	−1.49	12.71	21.4	4.7	4.82	1.188
Cr_0.5_	8.99	2495	−2.47	13.15	13.3	5.3	4.89	1.189

**Table 5:** Results of the tensile tests.

Temp.		R_b_ (MPa)	ε_b_ (%)	Temp.		R_b_ (MPa)	ε_b_ (%)
RT	Cr_0_	915	8.56	600 °C	Cr_0_	316	3.22
	Cr_0.25_	513	0.35		Cr_0.25_	236	0.04
	Cr_0.5_	261	0.1		Cr_0.5_	300	0.18

**Table 6:** Compositions of points 10 and 11 (at.%).

Point	Ti	V	Nb	Ta	Cr
10	81.20	4.41	7.75	4.95	1.68
11	90.28	1.32	4.86	2.32	1.22

**Table 7:** Composition of the oxidation layer in Figure 8 (at%, on behalf of Cr_0.5_ at 600 °C).

Point	O	Ti	V	Nb	Ta	Cr
12	62.03	3.70	13.31	12.02	8.16	0.78
13	69.77	2.12	4.17	15.83	8.11	-

## Data Availability

The original contributions presented in this study are included in the article. Further inquiries can be directed to the corresponding author.
